# Gen Z and Esports: Digitizing the Live Event Brand

**DOI:** 10.1007/978-3-030-65785-7_16

**Published:** 2020-11-28

**Authors:** Rebecca de Freitas

**Affiliations:** 1 Department for Informatics, Technical University of Munich, Garching bei München, Bayern Germany; 2 Smart Tourism Education Platform (STEP) College of Hotel and Tourism Management, Kyung Hee University, Seoul, Korea (Republic of); 3 Department of Tourism and Service Management, MODUL University Vienna, Vienna, Wien Austria; Georgetown University School of Continuing Studies, Washington, DC 20001 USA

**Keywords:** Esports, Event management, Gamification, Gen Z, Live events

## Abstract

As digitization converges with globalization, industries across the world establish new standards, platforms and audience engagement methods to delight consumers adjusting to CV19’s virtual space. Within the Tourism and Hospitality industry, gamification provides the events and meetings sector an opportunity to implement hybrid events at a level unseen before. Esports is the newest standard of gamification for hybrid, both live and virtual, events. However, within this new standard, there is a large knowledge gap among event organizers of how to execute an esport experience and why esports dominance is necessary to incorporate into hospitality and tourism models. Through understanding esports’ majority consumer, Gen Z, and accurately reflecting esports culture, event organizers will assist the tourism economy through prosperous esport events.

## Background

Defined as a multiplayer competition using video games through mobile PC and console platforms esports has transformed from a solitary niche subculture into a lucrative industry with an estimated market growth of $2.5 billion in revenue by the year 2025 [1]. Long gone are the days as a stereotypical refuge of nerdy hobbyists or introverted gamers playing PCs (web-based games) or consoles (i.e.: Nintendo Switch PlayStation and Xbox). In 2018 there were 737 major events. When combined these events generated “$54.7 million in ticket revenue” [2, 3]. Today esports can be a fully immersive three-dimensional video game experience in the event arena setting in front of fans or mobile game leisure individual play in the hands of the consumer (i.e.: games on tablets and smartphones).

When esports is presented before an audience it is a logistically complex event with digitally advanced and glamorous sponsors [4]. Needs such as hotel accommodations for esport tournaments personnel and the travel schedules of esport teams sponsors and fans create immense hospitality requirements. Tourism and Hospitality professionals provide venue familiarities deadline appreciation and logistical steps to guarantee a phenomenal esport experience. These same professionals provide airline bookings restaurant reservations and a destination marketing plan to support the circular economy growth of local esport events.

## Introduction

Riot Games’ League of Legends Championship Series (LCS) in the summer of 2019 is credited for contributing $5.44 million to the economy of the host city, Detroit, Michigan [5]. The 2019 LCS Summer Finals was held in Detroit’s Little Caesars Arena for 48 h, from August 24th–August 25th. During open business hours, the LCS was generating $226,666 an hour to the local Detroit economy. A majority of these business were hospitality driven; restaurants, hotels, casinos or travel bookings. Tourist hotspots, as Hangzhou and Raleigh, have advanced this model into larger more permanent investments. Anticipating the swell of esport enthusiasts, Hangzhou created an esports town complex to the cost of $280 million with 3.94 million square feet in 2018 [6]. Similarly, the Greater Raleigh Convention reported $1.45 million in direct economic impact resulting from R6 Raleigh Major at the Raleigh Convention Center [7]. With CV19 pivoting interest away from these expensive infrastructures’ capacity, it is important to understand esports’ young consumers and their disposable incomes.

When these young consumers, with growing disposable incomes, are matched with the projected revenue growth of $2.5 billion, the future of esport is a compelling business proposition. [1]. Esport’s perceived explosive monetary potential has led to a flood of non-esport corporations pursuing ways to successfully position themselves in the segment [8]. 59% of esports financials are generated from these corporate brands, private equity investors and venture capitalists. These entities do not have an esport governing body for support, to facilitate government relationships, or reliable broad data on their consumers preferences. An appropriate analogy would be similar to the NFL did not exist to supervise football in the United States or if Monaco was to plan the Grand Prix without Formula One Group’s regulations.

As a result, companies risk expending financial resources unwisely and ending up, like so many unseasoned gamers, getting “ganked”, or defeated in the game [9]. Additionally, there is little available research available on esports event’s economic impact, outside of the post event press releases from CVBs concluding the an esport events’s local effect.

Esports is not a swift way to generate revenue or to meet the needs of younger consumers. Esports is a digital lifestyle and a sustainable hospitality business opportunity. Esports will influence live events and tourism moving forward. The opportunity is for the event industry to understand the esport consumer, make well educated decisions and sustain the hospitality industry in the digital future leveraging esports as an economic platform for the segment.

### Purpose

The amalgamation of esports’ economic impact post CV19 and the digital model of Gen Z’s consumerism is palpable; to connect with the future Gen Z traveler, an event organizer’s digital message of esports must be clear, whether on behalf of the event itself or using a Destination Management Organization (DMO) platform. The combination of this emerging esport market and esports’ young demographic has propelled interested corporations to initiate as many esport events as possible to create audience retention and revenue. This marathon of events, endured by the organizers and other tourism professionals, is exacerbated because there is no consensual, governing esport body to mandate procedures or initiate structured esport event operations.

The challenge e facing the Tourism and Hospitality industry is how to capture and sustain the monetization opportunity of esports; in an environment lacking governance structure, where leading technology providers can greatly influence all aspects of the segment, and the young consumer responds to stimuli differently than historical patterns. Event organizers are uninformed when executing an esport event. The purpose of this research is to close the knowledge gap among event organizers regarding how to execute a Gen Z centered esport event among these challenges. Thus, why esports is necessary to incorporate into hospitality and tourism models. There is little accredited research available on esports’ economic impact within the tourism and hospitality vertical. Rather, research available is often a published whitepaper of a sales seeking organization or a press release from a CVB post event on the esport event’s impact. Consequently, the purpose of researching the esport consumer behavior patterns of the Gen Z demographic is to predict the esport event trends, and the potential economic impact on future hospitality and tourism business models.

### Theory

The goal of this research is to be transparent and transferrable for other tourism and event organizers to replicate the process when researching or executing an esport event. The theoretical framework is centered in researching Gen Z esport consumer trends, and effective esport event implementations in a post CV19 space. The belief that esports provide positive growth drives the data collection process.

This conceptual framework, will guide the reader through executive interviews and data provided by survey participants by emphasizing the consistencies found in the data collection which support effective esport event execution for the Gen Z consumer and positive growth in the hospitality sector of local economies. The objective of this esport event research is to demonstrate value so as to be considered for distribution among numerous organizations, including Meeting Planner International (MPI), Events Industry Council (EIC) and Professional Convention Management Association (PCMA) for possible inclusion in their respective Certified Meeting Professional (CMP) and Digital Event Strategist (DES) courses certification course.

## Methodology

This study is a mixed method approach. Data is synthesized from fifteen qualitative esport executive interviews to create a singular quantitative survey. The survey is circulated among a large esport professional network. Executive summaries from the fifteen interviews and applicable stats from the subject survey will inform esport event organizers and tourism professionals in executing an esport event for the Gen Z consumer. Our objective is to use our research to minimize the identified knowledge gap and to guide the tourism industry into the digital future post CV19.
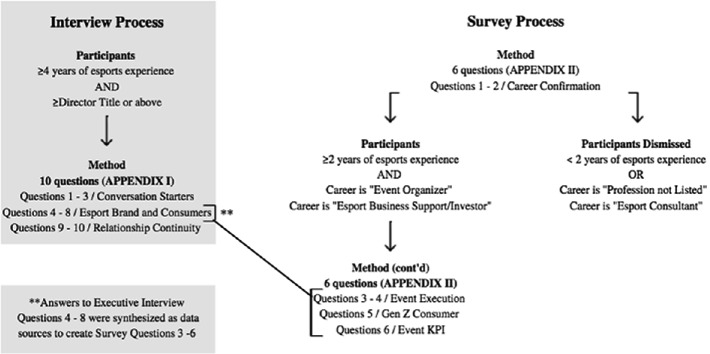


### Interview Participants

The data collection process was initiated by interviewing esport executives who possess a broad understanding, deep insight and forecasting knowledge of the industry. This is the primary source of data with a qualitative intent. Participants for GenZ and Esports: Digitizing the Live Event Brand executive interviews were individuals with a Director title or above and at least four years of experience in the esport space. The primary sources are the premiers in a range of organizations within the esport ecosystem; video game publishers, esport media outlets, digital event organizers, gaming investors, branding agencies and esport leagues/teams. There was no geographical limitation as to where these individuals lived or worked. Rather, the only constraint was the individual must have a Director title, or above, and have at least four years’ experience within the esport space.

The executives interviewed were each asked ten identical questions; the first three questions were conversation generators whose purpose was to create a welcoming and trusting dialogue between the student and the interviewer. This was followed by five questions regarding the esport event brand and consumer and then closing with survey continuity, an attempt to continue the relationship for research purposes. The exact interview questions posed are located in Appendix I.

The first interview was hosted on January 26th, 2020 and the fifteenth interview was completed in two months’ time on March 26th, 2020. As the executive interviews were completed, the student transcribed the interviews into a Microsoft excel software. The executives’ varying answers are transliterated as anonymous “data sources.” From this software, common themes were discovered from the data sources and developed into a six-question survey.

### Survey Participants

The second, quantitative component of this research is a survey amongst a select group sampling of esport professionals with at least two years of experience within the esport industry. This survey is a primary source of data with the objective to quantify numerically the solutions event organizers should utilize when hosting an esport event to position the event for success; achieving financial margins, effective branding and audience retention within the Gen Z demographic.

In addition, this survey pool of esport professionals’ places of employment mirror the organizations from the esport executives who participated in the preliminary interviews were selected. 9,000 survey participants were contacted through LinkedIn or Email with a survey link bringing the participants to the survey host site, Survey Monkey. This universe of 9,000 esport surveys and the sampling pool of data was developed from professional esports networking groups and recommendations of both individuals and groups from the executives who were interviewed.

The speed of monetization in the esport industry is overwhelming with many corporations racing to partake in the exciting industry. However, the speed of which certain corporations are entering the market leave little time to understand the true value proposition that esports provides the event and tourism space. There are several actors in the space who claim to have significant experience and expertise in esports when it is simply not true. Two questions at the beginning of the survey were to serve as vetting filters to select the most experienced participants with rich esport event knowledge. The first question was asking survey participants how many years they had been involved in esports. Survey participants with less than two years experiences were eliminated. The second question asked survey participants to provide their profession. Survey participants who answered either “profession not listed” or “esport consultant” were also eliminated. Candidates who listed their profession as event organizer or business support investor had their survey counted. In order to create a knowledgeable survey pool, it was necessary to eliminate those participants who could not clearly define their professional role in the esport ecosystem.

The result of this vetting was to focus on event organizers, esport investors and esport professionals who have had at least two years’ experience in the fairly young esport industry. The survey included four questions, intended to fit on one email page for the convenience of the survey participant. The questions relate to:

1. Esport Event Execution
2. The Gen Z Esport Consumer
3. Esport Brands’ Metric

Each question was created by synthesizing the primary source data from the executive interviews for common themes; event execution, consumer and esport brand, as mentioned. The exact survey questions are distributed survey findings are located in Fig. 4, 5, 6 and 7.

The survey was launched on March 30th, 2020 and was circulated over time among 9,000 esport professionals through April 20th. March 30th is a Monday and Mondays are proven to carry the highest engagement and response rate [10]. The survey period included 4 Monday’s. The survey software used to distribute the survey was Survey Monkey.

### Secondary Sources of Literature

Secondary sources are literature and bibliography’s references from which were selected from tourism and hospitality industry news articles, academic journals, market reports, published interviews and corporate white papers. The specific data obtained from these secondary, literary sources was used to either support or contradict the primary source data. Due to absence of esport governance and the overwhelming number of actors in the esport ecosystem, there are limited cohesive studies on live event esport organizing. This lack of quality reference literature material is a contributing factor to the knowledge gap existing among event organizers relative to esports.

Ironically, the esport industry itself is data dense; with Big Data, digital archives and player statistics yet, there have been limited studies on esport event organizing. These limited research opportunities are not unusual in a developing industry, but the lack of data makes drawing conclusions difficult. The industry’s explosive growth, its newness, lack of attributes similar to other sporting activities, and targeted demographic’s still developing disposable income stream make drawing conclusions fluid, until data becomes more reliable. As the problem statement states, in the intervening period, there are several esport knowledge gaps developing pertaining to audience retention, consumer reports and return on investments within the tourism and hospitality industry.

## Results

### Qualitative Executive Interview Summary

The qualitative approach summarizes the esport community’s voice from an executive level, framing what may occur in the next 3 to 5 years in the future. The ten questions, mentioned earlier and located in Appendix I, led to an open conversation with executives. As the executive interviews were completed, the recording of each interview, with the interviewee’s permission, is transcribed into an excel software. The executives’ varying answers are transliterated as anonymous “data sources”. Common themes (esport consumer, esport event execution and esport metrics) are discovered from the data sources and implemented into a four-question survey.

**Fig. 1.**
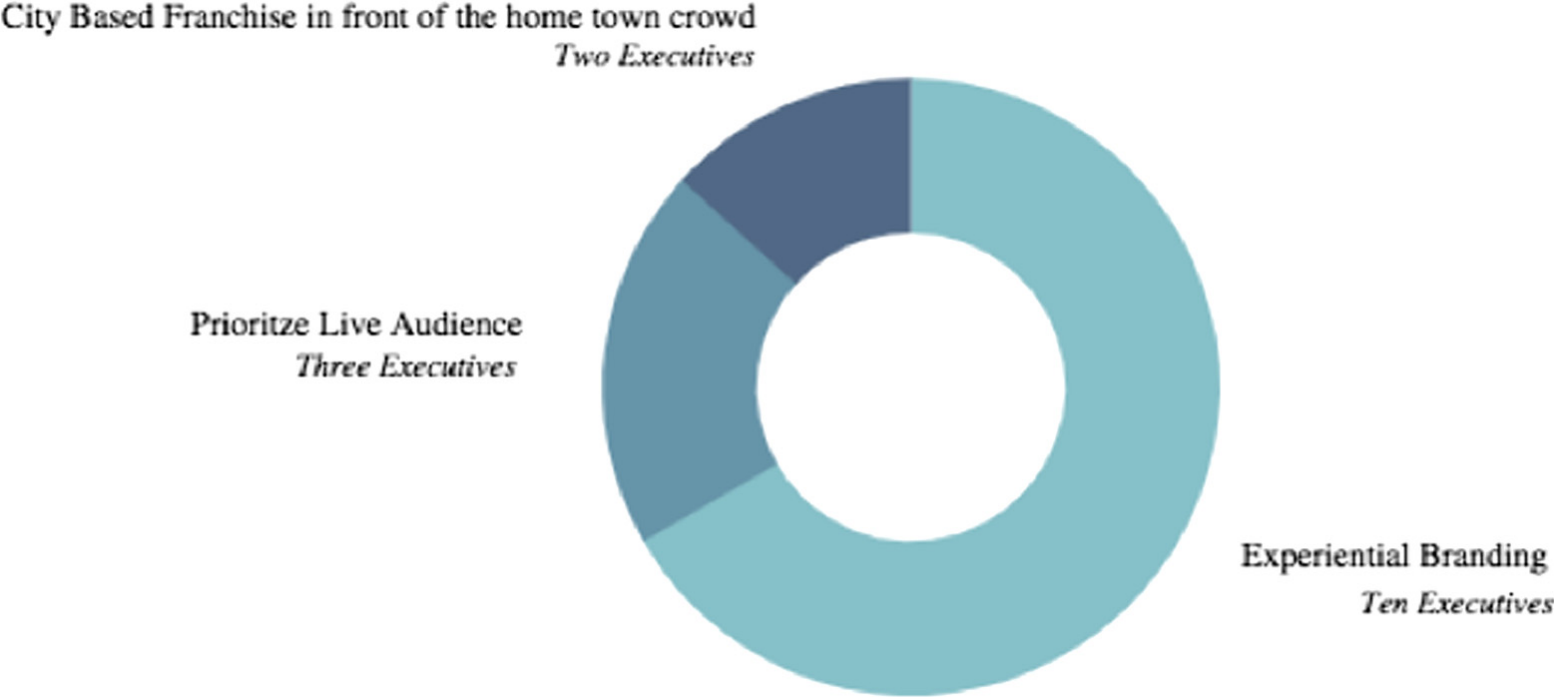
How do you recommend synthesizing a live event brand experience with a streaming audience simultaneously? Fifteen Executive Interviews to confirm.

When executives were asked for recommendations regarding how to present uniform branding across both live events and streaming event audiences (Fig. 1, following page), 67% (ten) of executives referred to live event experiential branding within the actual esport event. Thus, the final question in the survey was a direct result of this executive response. With the plethora of virtual platforms emerging post CV19, it is critical for event organizers and tourism professionals to choose the correct platform that prioritizes the live event esport experiential branding. Virtual event platforms have varied abilities such as live workshops, engaging chat features and webcasting yet, the foundation of the capabilities must be the live event esports experiential branding. For example, when executing an esport event the event organizers and tourism professionals should consider creating avatars that would be represented in the physical, live event model while streaming in the digital broadcast simultaneously. This avatar could be a sponsor’s mascot or a local concert hall that is providing music for the event.

**Fig. 2.**
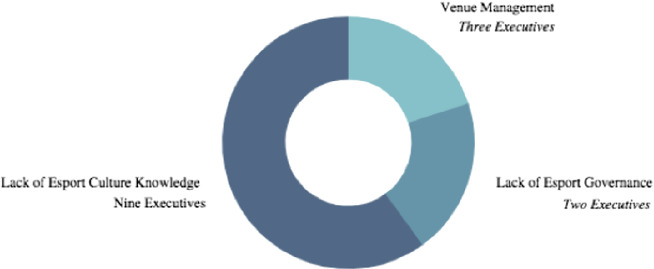
What are the challenges you foresee for event organizers who are becoming familiar with esports? Fifteen Executive Interviews to confirm.

The second question (Fig. 2, below) executives answered in the interviews was; what are the challenges you foresee for event organizers who are exploring or becoming familiar with esports as a business opportunity? A majority of executives, (9) referred to the lack of consistent data and lack of knowledge of the esport culture stipulating event organizer’s challenges. This became the basis of the third survey question; asking survey participants to clarify exactly what challenges they are facing. The survey participants’ answers to the third question were aggregated and presented so as to alleviate the lack of data plaguing the event organizers interested in esports.

The objective of this research is to demonstrate value so it may be included in the respective CMP and DES certification courses of, MPI, EIC, and PCMA. The data’s value is to shrink the knowledge gap of esport culture within the tourism industry and to frame an industry wide standard of event execution, in the absence of esport body governance.

**Fig. 3.**
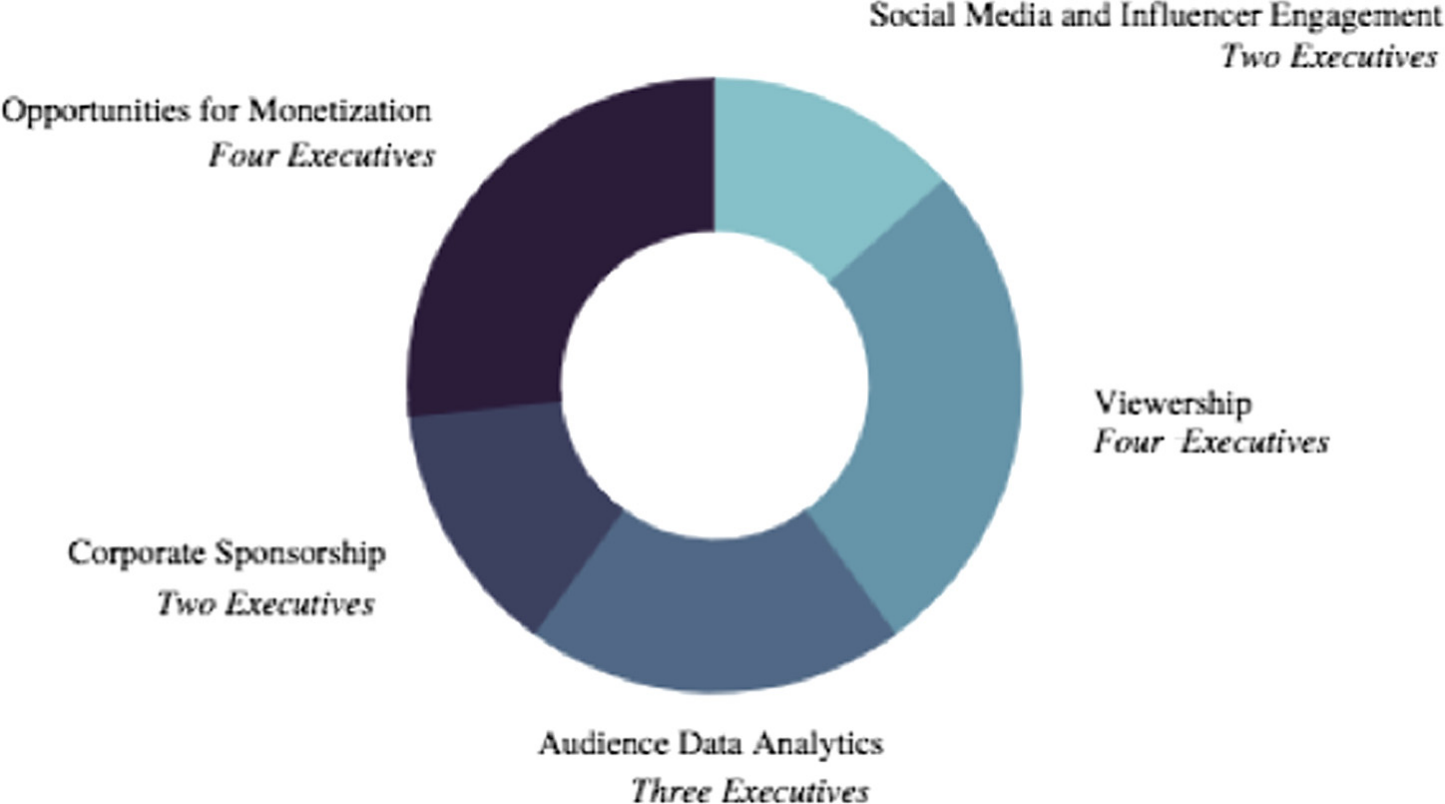
What are the metrics you prefer when approaching an esports project? Fifteen Executive Interviews to confirm.

Lastly, (Fig. 3, following page), what are the metrics you prefer when approaching an esport project? There was little variation between the five common answers. Without a reliable distinction of responses available, the five common answers were segmented into four of the survey questions. Whether the five commonalities were used as answer options or within the survey question body itself, this question proved to provide the widest array and most revealing trends of event organizers when approaching esport opportunities.

The encompassing opportunities of monetization and viewership can be reorganized through the direct (Corporate Sponsorship, below) and indirect revenue (Social Media and Influence Engagement, below) amalgamation of Audience Data Analytics. An example of direct revenue through corporate sponsorship at an esport event would be a creating an “Experience Bundle” for a Gen Z consumer that would include an Instagram Live (IG Live) chat with their favorite gamer, tickets to the esport event and passes to local restaurant sponsor to enjoy after the esport event has passed. Additionally, to expand viewership, indirect revenue and audience data analytics event organizers must promote live event esports on social media channels. Utilizing famous esport influencers or local thought leaders can encourage esport event attendance and ticket purchase.

### Quantitative Survey Results

The quantitative research survey provides numbers to highlight which solutions are needed to close the esport knowledge gap among event organizers and tourism professionals. As completed, the survey’s response is presented in a graph format that represents the survey’s pool response numerically. The survey’s results present the following data points.

**Fig. 4.**
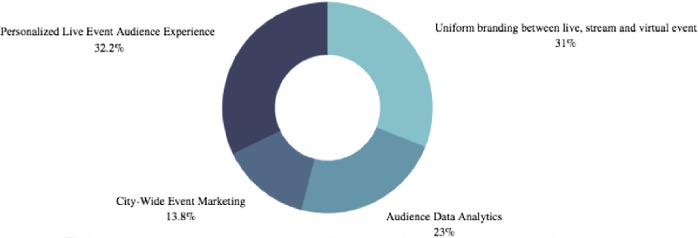
For an event sponsor, what is the most important task to be executed when planning an esports event? One hundred survey participants selected from nine thousand survey pool.

The first question (Fig. 4, following page) directly asked the survey participants to vote for the singular most important component of an esport event. Personalized experiences for the live event audience ranked first with over 32% of survey participants voting this as the most important esport event task to executed successfully. However, trailing 1.2% behind was the duty of presenting uniformed branding between both the live esport event audience and streaming audiences.

As the survey participants were fairly young, 70% under the age of 45, it is paramount that experiential branding is spotlighted. As mentioned earlier, the Gen Z consumer favors experience over material items and with event organizers at the helm sharing in the experiential thought process, client and host are aligned. Keep in mind, GenZ consumers prioritize experience over material goods, socio-economic progressive policies and loyalty to an esport athlete rather an esport team. This solidified the solution to the problem of esports’ knowledge gap amongst event organizers: experiential branding and personalized experiences.

As the largest demographic, consumers between the ages of 18–34, are credited as 62% of the esport market [11]. The Gen Z demographic is the first demographic to grow up with a world online. It is deemed digitally fluent and can navigate the internet and social media with ease. Through this ease of navigation and familiarity of the internet, it is cited that 85% of Gen Z individuals engage with branded content on social media [11].

**Fig. 5.**
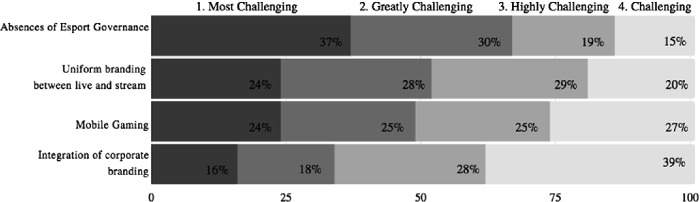
Rank the following, starting with 1, what is the greatest challenge esport event organizers will face in 2021? One hundred survey participants selected from nine thousand survey pool.

The third question (Fig. 5, following page) was also a ranking analysis; what is the greatest challenge event organizers will face in 2021? The survey participants ranked the absence of an esport governing body to be the greatest challenge in esport event organization, with 37% of survey participants voting in favor of this.

This response presented an anomaly for there was a tie for the second challenge event organizers will face. 24% of survey participants voted that mobile gaming is greatest challenge facing esports, while an additional 24% believed presenting a uniform brand experience between both the live event and streaming event. This question confirmed the problem statement and the industry’s need for the esport knowledge gap to be closed as there is no esport governance to guide and provide structure to such growth.

This question provided surprising results as mobile gaming emerged as a great concern amongst the survey participants. In North America, mobile gaming is often dismissed as a leisurely past time and not included in the professional realm of competitive esports that are played on PC or consoles.

For event organizers and DMOs to be concerned that mobile gaming will overtake traditional esports is prudent. e. Mobile gaming is easily accessible via cell phones or iPads. This accessibility may eliminate the need for event space gatherings to observe video game play. However, our view is while emboldened by the current COVID 19 pandemic, this challenge will occur overtime. In APAC, mobile is escalating and the mobile platform has improved to run high quality games for PCs or consoles. Also, in this region, from 2016 to 2018, a game called Honor of Kings went viral and became the most popular game in China. In 2018, the King Pro League (Honor of Kings) Spring tournament sold out Shanghai’s Mercedes-Benz Arena, with a 18,000 capacity, within minutes of ticket sales opening [2]. Research encourages readers to see mobile gaming is not a challenge to traditional esports so much as it is a new, exciting platform for event organizers to implement.

**Fig. 6.**
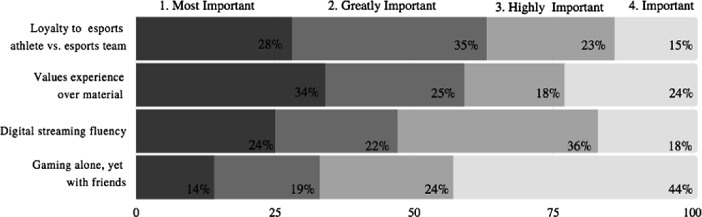
Rank the following, starting with 1, what is the most important attribute of the youngest esport consumer, Gen Z (8–24)? One hundred survey participants selected from nine thousand survey pool.

The third question, (Fig. 6, below), survey participants were asked to rank the most important attribute of the Gen Z Esport consumer, the Gen Z’s preference to be loyal to an esport athlete rather than an esport team was superior. 68% of survey participants voted Gen Z’s esport athlete loyalty to be either the most important second most important attribute. This was followed in a close second by Gen Z’s value of experience over material items, with 59% of survey participants voting the experiential presence to be either the most important or second most important of the Gen Z Esport audience

This is key for event organizers who handle sponsorship or experiential branding accounts. These fervent fans are likely to be weekly streamers who follow and make donations to their favorite esport athlete or fellow streamer. There were instances when game publishers released streaming content that feature esport celebrities playing each or top-level amateur players (Eg: Fortnite Summer Skirmish). Transitioning the esport fan’s enthusiasm from online to live events will be executed through fan vs. influencer experiences.

For example, event organizers and DMOs should consider the possibility of providing a “fervent fan” tour of the esport facilities and arenas such as the Philadelphia Fusion arena. Furthermore, inviting the fans to play side by side with the esport athletes themselves within the complex. The experience of connecting with an esport influencer or celebrity will solidify a Gen Z’s interest in attending a live event.

**Fig. 7.**
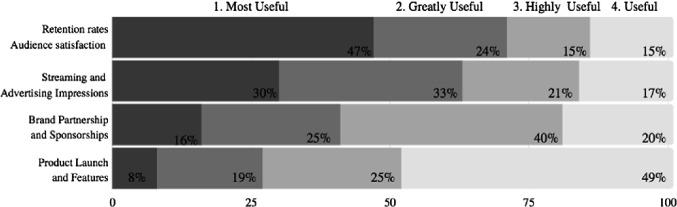
Rank the following, starting with 1, what is the most useful source of KPI (Key Performance Indicator) for esports events? One hundred survey participants selected from nine thousand survey pool.

The second survey (Fig. 7, below) question presented a wider variance of survey responses. The question inquired as to what the most useful Key Performance Indicator (KPI) is and how it should be ranked between the “most useful” to “useful”. 47% of survey respondents revealed that esport retention rates and audience satisfaction is the singular most useful form of KPI. The second most useful KPI was deemed to be esport streaming and advertising impressions by 30% of the survey participants.

The retention rate factor listed in Fig. 7 is essential. Many esport leagues operate utilizing local teams and are planning the construction of local esport venues. Local fans and consumers are the retention demographic demanded because of their geographical proximity to the esport brand and, if a great live event experience, guaranteed return trips. Localization of esports will be able to include local vendors, DMOs, community sponsors and create a familiar environment esport fans can repeatedly return too.

## Conclusion

Finding a way, to integrate esport content and the consumer is challenging. Yet, corporations heeding to the consumer base’ preferences [12, 13]. and manifesting authenticity is clearly demanded by esport consumers. For example, questions were raised when The Overwatch League, the brainchild of Activation BlizzCon was cited as floating $300 million valuations for a $25 million revenue. It was mysterious because the valuations were twelve times larger than the revenue and there was no mention of forecasted profit. Although, capitalists want to find those opportunities to get advantageous ROI’s, it was the esport fanbase who protested and felt deceived [8].

As authenticity drives the esport world, the student chose to approach data from both a qualitative approach and a quantitative approach. With both gygies implemented there would be an executive voice heard, qualitatively, and an event management opinion heard, quantitatively. Thus, emerging a more well-rounded report of data from different roles within the esport live event community.

By gathering data, both quantitative and qualitative, and then analyzing the data along with primary and secondary sources, a broad perspective and deep-dive insight of esport emerges. The data collection will ensure applicable stats (survey) and executive summaries (executive interviews) for those readers who are making decisions for an esport event. The student emphasizes that by connecting with the streaming consumer, event organizers can meet and influence consumer decisions at their esport passion points; [14] Through diversifying digital brand options (fashion, lifestyle, luxury, food and beverage, entertainment, etc.) that offer esport fans something in addition to the game, consumer retention is guaranteed [15].

As experiential branding and the live event esport experiences strive to wow and delight consumers, it is paramount to recognize the consumer’s willingness to be enchanted. A post event survey distributed by the Consumer Electronic Show (CES) revealed how event organizers on the CES team utilized the consumer’s openness to be transported to another world by providing virtual reality [15]. This virtual reality was an incredibly realistic and undisturbed journey into an immersion room that could not be achieved at home. Consumers who entered the immersion were enthralled and provided rave reviews of their CES event experience.

The arc is identical as event organizers find a way to integrate the esport content with the experience infatuated esport consumer. Two op-ed pieces from the events industry confirmed that by listening to the first consumer base’ preferences [13, 16] event organizers will legitimize esport and provide the authentic connection desired at esport events. On-site solutions for event organizers to achieve this bridge into the digital include designating digital meeting spaces or launching event apps to enhance the event’s immersive experience [17]. The streamline of digital approaches leaves more time for creativity and event organizers can utilize this creativity to create Q&A chat functions; thus, see what questions the audience might have or give the platform for audience members to share their own expertise or event feedback.

Associations and organizations have been considering when or if to invest in esports. That question is no longer relevant as esports is a booming and mature industry saturated with corporations. The associations and organizations need to ask now how to expand their event programs into the esport business.

## Appendix I

1. What is your favorite video game?
2. What has been the premier esport experience you’ve encountered?
3. How many years have you been working along esports professionally?
4. How do you recommend synthesizing a live audience esport brand experience while streaming online simultaneously?
5. What are the challenges you foresee for event organizers who becoming familiar with esports?
6. What strategies do you implement to create a personalized esport experience for attendees?
7. In your opinion, what are the top metrics that sponsorships are looking for?
8. How do you use data to personalize and enhance the consumer experience at an esport event?
9. Will you be willing to participate in a survey post interview?
10. Are you in a position to circulate the identical survey to other five other esport professionals?
